# Randomized, placebo-controlled, adjunctive study of armodafinil for bipolar I depression: implications of novel drug design and heterogeneity of concurrent bipolar maintenance treatments

**DOI:** 10.1186/s40345-015-0034-0

**Published:** 2015-09-02

**Authors:** Mark A Frye, Jess Amchin, Michael Bauer, Caleb Adler, Ronghua Yang, Terence A Ketter

**Affiliations:** Department of Psychiatry, Mayo Clinic, 200 First Street SW, Rochester, MN 55901 USA; Teva Pharmaceuticals, Frazer, PA USA; University Hospital Carl Gustav Carus, Technische Universität Dresden, Dresden, Germany; University of Cincinnati, Cincinnati, OH USA; Stanford University, Stanford, CA USA

**Keywords:** Armodafinil, Bipolar I disorder, Major depressive episode

## Abstract

**Background:**

Some, but not all, prior investigations suggest armodafinil may have utility as an adjunctive treatment in bipolar I depression.

**Methods:**

Multicenter, randomized, double-blind study in patients aged 18 to 65 years experiencing a depressive episode despite maintenance therapy for bipolar I disorder. Patients were randomized to receive adjunctive armodafinil 150 mg/day or adjunctive placebo for 8 weeks. Primary efficacy outcome was change from baseline in 30-Item Inventory of Depressive Symptomatology–Clinician-Rated (IDS-C_30_) total score at week 8. Safety and tolerability were monitored.

**Results:**

Of 656 patients screened, 399 were randomized, of whom 308 (77 %) were taking a protocol-allowed mood stabilizer as monotherapy. The primary efficacy outcome did not reach statistical significance; however, several secondary efficacy outcomes demonstrated statistically significant advantages for adjunctive armodafinil (*n* = 197) over adjunctive placebo (*n* = 196), including Clinical Global Impression of Severity of Illness for depression (weeks 6, 8, and endpoint; all *P* < 0.05), Global Assessment of Functioning (weeks 4, 8, and endpoint; all *P* < 0.02), IDS-C_30_ remitter rates (week 8 and endpoint; both *P* < 0.02), and mean change from baseline in IDS-C_30_ total score at week 7 (*P* < 0.05). Adjunctive armodafinil and adjunctive placebo were generally well tolerated. Although adjunctive armodafinil compared with adjunctive placebo yielded a higher headache rate (15 vs 8 %), it yielded similar (generally favorably low) rates of all-cause discontinuation (16 vs 16 %), adverse event discontinuation (4 vs 5 %), nausea (6 vs 4 %), ≥7 % weight gain (2 vs 5 %), anxiety (4 vs 3 %), insomnia (3 vs 2 %), sedation/somnolence (1 vs 1 %), and hypomania (0 vs <1 %).

**Conclusions:**

In this study, adjunctive armodafinil compared with adjunctive placebo in bipolar I depression did not separate in the primary efficacy outcome but demonstrated advantages for several secondary efficacy outcomes and was generally well tolerated. Additional research is warranted and necessary to better identify clinical predictors (e.g., atypical depressive symptoms, specific anti-manic/mood-stabilizing agents used) that would provide optimized, individualized therapeutics for bipolar depression.

**Trial registration:**

ClinicalTrials.gov: NCT01305408

## Background

Bipolar disorder is a recurrent and debilitating illness that affects approximately 2.6 % of the adult population (Kessler et al., 2005), with approximately 1 % of the population having bipolar I disorder (Merikangas et al., 2007). Depression is the predominant and prevailing symptomatic illness phase. For example, in bipolar I disorder, depression accounts for at least three times more time ill than mania (Judd et al., 2002; Kupka et al., 2007). Depressive episodes are associated with more disability, comorbidity, and functional and occupational deficits than manic episodes; moreover, the depressive phase is associated with an increased risk of recurrence of mood symptoms and rates of suicide (Bauer et al., 2001; Calabrese et al., 2004; Merikangas et al., 2007; Merikangas et al., 2011). In clinical settings, adjunctive treatments may be added to maintenance therapies when mood symptoms recur, but the clinical evidence base is limited in this type of therapeutic intervention. Only one agent, lurasidone, has been approved by the United States Food and Drug Administration (FDA) as adjunctive treatment, combined with either lithium or valproate for acute bipolar I depression (Latuda [package insert], 2013). FDA-approved treatments for acute bipolar I depression include the olanzapine/fluoxetine combination, quetiapine monotherapy, and lurasidone. Investigations of aripiprazole monotherapy (Thase et al., 2008) and ziprasidone, as both monotherapy and adjunctive therapy (Lombardo et al., 2012; Sachs et al., 2011), failed to provide significant improvements in depressive symptoms compared with placebo in bipolar I depression. Clearly, given the morbidity of bipolar depression, more treatment options are needed.

Armodafinil (*R*-modafinil) is a wakefulness-promoting low-affinity dopamine transport inhibitor that is currently approved in the USA for the treatment of excessive sleepiness associated with shift work disorder, narcolepsy, and obstructive sleep apnea (Nuvigil [package insert], 2013). Preliminary research on modafinil and armodafinil suggested potential benefit in mood disorders (Calabrese et al., 2010; Calabrese et al., 2014; Frye et al., 2007).

An initial phase 3 randomized, placebo-controlled study of adjunctive armodafinil 150 mg/day demonstrated a significant benefit in bipolar I depression on the primary outcome (mean change from baseline in the 30-Item Inventory of Depressive Symptomatology–Clinician-Rated (IDS-C_30_) total score) (Rush et al., 2000) in comparison with placebo (*P* < 0.01), as well as some secondary outcomes (Calabrese et al., 2014). In contrast, a second, similarly designed phase 3 study found that adjunctive armodafinil 150 mg/day provided numerically, but not statistically significantly, greater improvement in bipolar I depression than placebo on the same primary outcome (Ketter et al., 2015).

The current study (ClinicalTrials.gov, study identifier NCT01305408) was performed to further investigate the efficacy, safety, and tolerability of adjunctive armodafinil use in bipolar I depression. As in prior investigations, this study permitted adjunctive armodafinil or adjunctive placebo to be combined with a broad array of ongoing bipolar disorder maintenance treatments, in conformity with the availability of many such choices in clinical practice, providing the strength of increased generalizability, albeit with the potential limitation of decreased assay sensitivity. This study, unlike prior studies, included quetiapine as one of the permitted maintenance therapies, further contributing to this study’s generalizability and relevance to clinical practice. As in prior studies, efficacy was assessed by the mean change from baseline in IDS-C_30_ total score.

## Methods

This phase 3, 8-week, randomized, double-blind, placebo-controlled, parallel-group, fixed-dosage, multicenter study was conducted at 84 centers in 13 countries across 4 regions. Patients who were experiencing a major depressive episode, despite stable doses of protocol-allowed “mood stabilizers” (lithium and certain anticonvulsants and antipsychotics) for the treatment of bipolar I disorder, were randomly assigned to adjunctive armodafinil 150 mg/day or matching placebo in a 1:1 ratio. The 150-mg dose of armodafinil was selected based on efficacy and tolerability data from previous clinical trials. Randomization was stratified on the basis of concomitant mood stabilizers being taken (lithium, anticonvulsants, and antipsychotics). If a patient was taking more than one of these medications at randomization, the patient was assigned to the category of the medication of longest duration at the discretion of the investigator. Randomization was also stratified by region of the world (Region 1, the USA and Canada; Region 2, Armenia, Azerbaijan, Belarus, Bulgaria, Georgia, Russia, Ukraine, Kyrgyzstan, Mongolia, Uzbekistan, Albania, Bosnia, Herzegovina, Croatia, Cyprus, Czech Republic, Greece, Hungary, Poland, Serbia, Slovakia, Slovenia, Republic of Macedonia, and Turkey; Region 3, Andorra, Austria, Australia, Belgium, Denmark, Finland, France, Germany, Greece, Iceland, Ireland, Italy, Liechtenstein, Luxembourg, Malta, Monaco, the Netherlands, Norway, Portugal, San Marino, Spain, Sweden, Switzerland, the UK, and Vatican City; Region 4, rest of the world).

### Ethics, consent, and permissions

The study was conducted in accordance with the International Conference on Harmonisation’s Guideline for Good Clinical Practice, and the study protocol and amendments were approved by the independent ethics committee/institutional review board at each participating center. Written informed consent was obtained from each patient before screening.

### Participants

Inclusion and exclusion criteria were similar to those reported in detail for the previous two studies (Calabrese et al., 2014; Ketter et al., 2015). Specifically, patients were aged 18 to 65 years and had bipolar I disorder with current non-psychotic depression according to the *Diagnostic and Statistical Manual of Mental Disorders* (Fourth Edition, Text Revision; DSM-IV-TR) criteria (American Psychiatric Association, 2000), as determined by the Structured Clinical Interview for DSM-IV, Clinical Trials (SCID-CT) (First et al., 2007). Patients were required to have had ≥1 previous manic or mixed episode, which resulted in functional impairment that was treated (or should have been treated) with a protocol-allowed mood stabilizer. Patients could not have had >6 mood episodes in the prior year and their current depressive episode must have started ≥2 weeks but ≤12 months prior to the screening visit and occurred despite taking stable doses of one or two mood stabilizers, defined specifically as lithium, valproate, lamotrigine, olanzapine, quetiapine, aripiprazole, risperidone, or ziprasidone (ziprasidone only in combination with lithium, valproate, or lamotrigine). In patients taking two mood stabilizers, one was required to be lithium, valproate, or lamotrigine. Medications known to induce CYP3A4/5, such as carbamazepine, were not permitted within 14 days before the baseline visit or during the study. Furthermore, onset of the current depressive episode had to be ≥8 weeks after resolution of any previous mood episode. Concomitant antidepressant use was not allowed within 14 days or five half-lives before study entry or during the study. This exclusion was exercised to minimize the confounding of any effects seen regarding adjunctive drug–placebo differences in bipolar I depressive symptom improvement with armodafinil.

Patients were required to have had screening and baseline 16-Item Quick Inventory of Depressive Symptomatology–Clinician-Rated (QIDS-C_16_) (Rush et al., 2003) scores ≥13, Clinical Global Impression of Severity of Illness (CGI-S) for depression score ≥4, Young Mania Rating Scale (YMRS) (Young et al., 1978) total score ≤10, and YMRS scores of 0 or 1 on items 1 to 3. Patients were also required to be in good health based on a physical examination, electrocardiogram, and laboratory studies.

Patients were excluded if they had other Axis I disorders within 6 months of screening that were the focus of treatment, or Axis II disorders of concern (borderline, antisocial, or other personality disorders that could impact conduct of the study), a history of alcohol or substance abuse or dependence (with the exception of nicotine dependence) within 3 months of the screening visit or during the screening period, current psychotic symptoms or psychosis within 4 weeks of screening, active suicidal ideation or history of significant suicidal behaviors, score of ≥2 on item 18 of the IDS-C_30_, Hamilton Anxiety Scale (HAM-A) (Hamilton Anxiety Rating Scale, 2011) total score ≥17 at baseline, or a history of clinically significant cutaneous drug or hypersensitivity reactions.

### Assessments

The primary efficacy assessment was the mean change from baseline assessed at week 8 in total IDS-C_30_ score for adjunctive armodafinil 150 mg/day versus adjunctive placebo as analyzed by mixed-model repeated measures (MMRM). Secondary efficacy assessments included mean change from baseline in the IDS-C_30_, QIDS-C_16_, and CGI-S, as well as IDS-C_30_ response (≥50 % reduction from baseline in total score), IDS-C_30_ remission (final IDS-C_30_ score ≤11) rates, and CGI-S response (decrease ≥2 points in severity from baseline) rate, all assessed at weeks 1, 2, 4, 6, 7, and 8 (or early termination), as well as mean change from baseline in the Global Assessment of Functioning (GAF), assessed at weeks 4 and 8, or early termination.

Safety assessments included mean change from baseline in the YMRS and the Columbia-Suicide Severity Rating Scale-Since Last Visit (C-SSRS-SLV) (Posner et al., 2009) at weeks 1, 2, 4, 6, 7, and 8 (or early termination), mean change from baseline in the HAM-A and Insomnia Severity Index (ISI) (Bastien et al., 2001) at weeks 4 and 8 (or early termination), as well as collection of adverse events (AEs) and serious AEs (SAEs), vital signs, and laboratory studies.

### Statistical analysis

Sample-size calculations were based on IDS-C_30_ total score and used estimates of variability obtained from previous phase 2 and 3 results for armodafinil and modafinil investigations in bipolar I depression (Calabrese et al., 2010; Calabrese et al., 2014; Frye et al., 2007). Target enrollment was 370 patients (185 patients for each of adjunctive armodafinil 150 mg/day and adjunctive placebo) to ensure that at least 332 patients (166 per group) were evaluable for efficacy, providing 85 % power to detect a mean between-group difference of 4 points in the change from baseline in IDS-C_30_ total score (assuming a standard deviation of 12.1). Data from 393 and 398 patients were analyzed for efficacy and safety, respectively.

Patients receiving ≥1 dose of study drug were analyzed for safety (safety analysis set), and patients in the safety analysis set who had ≥1 post-baseline IDS-C_30_ efficacy assessment were analyzed for efficacy (full analysis set). For the primary outcome, IDS-C_30_ total score was analyzed using MMRM as previously described (Calabrese et al., 2014). Continuous secondary efficacy variables were analyzed using analysis of variance and categorical secondary variables were analyzed by Cochran-Mantel-Haenszel test, as previously described (Calabrese et al., 2014). Safety and tolerability were monitored throughout the study.

## Results and discussion

### Participants

**Fig. 1:**
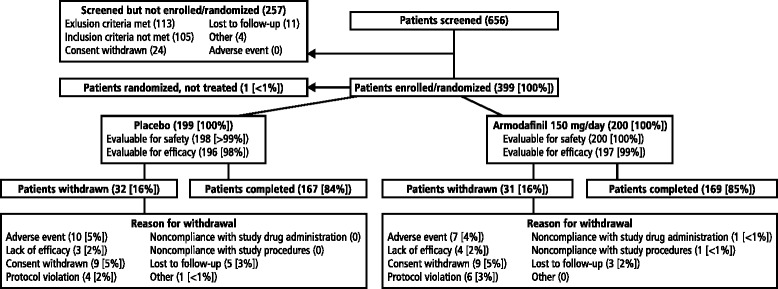
Patient flow

Of the 656 patients with bipolar I depression who were screened, 399 were enrolled; 200 were randomized to receive adjunctive armodafinil 150 mg/day and 199 were randomized to receive adjunctive placebo (Fig. 1). Baseline demographic and clinical characteristics were statistically similar between treatment groups (Table 1). The efficacy analysis included a total of 393 patients (adjunctive armodafinil 150 mg/day, *n* = 197; adjunctive placebo, *n* = 196); the safety analysis set included 398 patients (adjunctive armodafinil 150 mg/day, *n* = 200; adjunctive placebo, *n* = 198). A total of 63 (16 %) patients withdrew from the study (31 (16 %) receiving adjunctive armodafinil and 32 (16 %) receiving adjunctive placebo). At baseline, most patients (*n* = 308, 77 %) were taking only one mood stabilizer/antipsychotic. Valproate, lamotrigine, and risperidone were the most common mood stabilizer/antipsychotic treatments taken as monotherapy (Table 2). At baseline, depression scores were consistent with moderate to severe depression and were statistically similar between treatment groups.

**Table 1:** Baseline demographics and clinical characteristics

Characteristic	Placebo *n* = 199	Armodafinil 150 mg/day *n* = 200
Age, years, mean (SD)	43.7 (11.6)	45.3 (11.3)
Males, *n* (%)	78 (39)	80 (40)
Race, *n* (%)		
White	176 (88)	182 (91)
Black	16 (8)	14 (7)
Other	7 (4)	4 (2)
Weight, kg, mean (SD)	81.2 (17.5)	80.7 (17.5)
IDS-C_30_ total score, mean (SD)	43.3 (7.7)	42.4 (7.7)
QIDS-C_16_ total score, mean (SD)	16.8 (2.7)	16.1 (2.7)
CGI-S score, mean (SD)	4.6 (0.6)	4.6 (0.7)
GAF score, mean (SD)	54.8 (6.7)	53.6 (8.9)
C-SSRS		
Suicidal behavior, *n* (%)		
Suicidal behavior, actual attempt	20 (10)	21 (11)
Non-suicidal self-injurious behavior	7 (4)	6 (3)
Interrupted attempt	3 (2)	2 (1)
Aborted attempt	4 (2)	4 (2)
Suicidal behavior	4 (2)	4 (2)
Preparatory acts or behavior	5 (3)	6 (3)
Suicidal ideation, *n* (%)		
Suicidal ideation, wish to be dead	27 (14)	37 (19)
Non-specific active suicidal thoughts	16 (8)	24 (12)
Any methods (no plan) without intent to act	12 (6)	20 (10)
Some intent to act without specific plan	9 (5)	14 (7)
Specific plan and intent	10 (5)	15 (8)
YMRS total score, mean (SD)	3.7 (2.1)	3.7 (2.2)
HAM-A total score, mean (SD)	12.6 (2.8)	12.5 (2.9)
ISI total score, mean (SD)	16.1 (5.2)	15.7 (5.5)

*CGI-S* Clinical Global Impression of Severity of Illness, *C-SSRS-SLV* Columbia-Suicide Severity Rating Scale-Since Last Visit, *GAF* Global Assessment of Functioning, *HAM-A* Hamilton Anxiety Scale, *IDS-C*_*30*_ 30-Item Inventory of Depressive Symptomatology–Clinician-Rated, *ISI* Insomnia Severity Index, *QIDS-C*_*16*_ 16-Item Quick Inventory of Depressive Symptomatology–Clinician-Rated, *SD* standard deviation, *YMRS* Young Mania Rating Scale

**Table 2:** Concomitant mood stabilizers

Mood stabilizer at baseline, *n* (%)	Placebo *n* = 199	Armodafinil 150 mg/day *n* = 200
Patients taking one mood stabilizer	150 (75)	158 (79)
Aripiprazole	16 (8)	9 (5)
Lamotrigine	26 (13)	28 (14)
Lithium	12 (6)	18 (9)
Olanzapine	23 (12)	25 (13)
Quetiapine	18 (9)	26 (13)
Risperidone	29 (15)	24 (12)
Valproic acid	26 (13)	28 (14)
Patients taking two mood stabilizers	45 (23)	38 (19)
Aripiprazole + lamotrigine	4 (2)	1 (<1)
Lamotrigine + quetiapine	7 (4)	1 (<1)
Lamotrigine + ziprasidone	1 (<1)	1 (<1)
Lithium + aripiprazole	1 (<1)	0
Lithium + lamotrigine	3 (2)	2 (1)
Lithium + olanzapine	2 (1)	0
Lithium + quetiapine	4 (2)	3 (2)
Lithium + risperidone	1 (<1)	1 (<1)
Lithium + valproic acid	2 (1)	1 (<1)
Lithium + ziprasidone	3 (2)	1 (<1)
Olanzapine + lamotrigine	1 (<1)	1 (<1)
Olanzapine + quetiapine	1 (<1)	0
Valproic acid + aripiprazole	0	2 (1)
Valproic acid + lamotrigine	2 (1)	0
Valproic acid + olanzapine	5 (3)	8 (4)
Valproic acid + quetiapine	5 (3)	9 (5)
Valproic acid + risperidone	3 (2)	5 (3)
Valproic acid + ziprasidone	0	2 (1)
Patients taking three mood stabilizers	1 (<1)	3 (2)
Lithium + lamotrigine + ziprasidone	0	1 (<1)
Valproic acid + aripiprazole + risperidone	0	1 (<1)
Valproic acid + olanzapine + lamotrigine	0	1 (<1)
Valproic acid + risperidone + quetiapine	1 (<1)	0
Patients with mood stabilizer unknown	3 (2)	1 (<1)

A total of 17 patients discontinued early due to AEs (adjunctive armodafinil 150 mg/day, 7/200 (4 %); adjunctive placebo, 10/199 (5 %)). Anxiety and bipolar I disorder were the only AEs that caused discontinuation in >1 patient; anxiety led to treatment discontinuation in 3 (2 %) of those treated with adjunctive armodafinil and 0 treated with adjunctive placebo; 2 patients taking adjunctive armodafinil and 0 patients taking adjunctive placebo discontinued due to bipolar I disorder.

### Efficacy

#### Primary efficacy

Baseline mean IDS-C_30_ scores were 42.4 in the adjunctive armodafinil group and 43.3 in the adjunctive placebo group. The least-square (LS) mean and standard error of the LS mean (SEM) change from baseline to week 8 on the IDS-C_30_ (primary efficacy parameter) for armodafinil versus placebo (−20.8 ± 0.99 vs −19.4 ± 0.99) in the adjunctive treatment of bipolar I depression was not statistically significant (*P* = 0.27).

#### Secondary efficacy

**Fig. 2:**
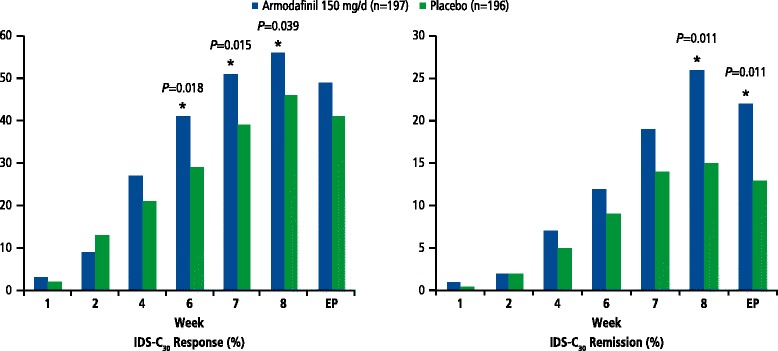
IDS-C_30_ response and remission. IDS-C_30_ = 30-Item Inventory of Depressive Symptomatology–Clinician-Rated

Although not adjusted for multiple comparisons, several secondary efficacy outcomes suggested advantages in favor of adjunctive armodafinil (Table 3). Specifically, statistically significant differences for the IDS-C_30_ secondary variables in favor of adjunctive armodafinil 150 mg/day over adjunctive placebo included proportion of IDS-C_30_ responders at week 6 (41 vs 29 %, *P* = 0.018), week 7 (51 vs 39 %, *P* = 0.015), and week 8 (56 vs 46 %, *P* = 0.039) (Fig. 2, left); proportion of IDS-C_30_ remitters at week 8 (26 vs 15 %, *P* = 0.011) and endpoint (22 vs 13 %, *P* = 0.011) (Fig. 2, right); and mean change from baseline in IDS-C_30_ total score at week 7 (*P* < 0.05). In addition, statistically significant differences in favor of adjunctive armodafinil were observed for the mean change from baseline in CGI-S rating for depression at week 6 (*P* < 0.03), week 8 (*P* < 0.02), and endpoint (*P* < 0.04) and in mean change from baseline in GAF scores, indicating improvement in patient functioning, at week 4 (*P* < 0.02), week 8 (*P* < 0.002), and endpoint (*P* < 0.01).

**Table 3:** Secondary efficacy parameters, full analysis set

Time point, statistic	Placebo *n* = 196	Armodafinil 150 mg/day *n* = 197	*P* value
LSM change from baseline in IDS-C_30_ total score			
Week 1	−6.1	−5.5	0.3025
Week 2	−10.4	−9.3	0.1940
Week 4	−12.3	−12.5	0.8481
Week 6	−14.2	−16.1	0.0926
Week 7*	*−16.0*	*−18.3*	*0.0492*
Week 8	−17.7	−19.6	0.1174
Endpoint	−18.3	−19.5	0.3526
LSM change from baseline in QIDS-C_16_ total score			
Week 1	−2.6	−2.4	0.3858
Week 2*	*−4.5*	*−3.8*	*0.0387*
Week 4	−5.2	−5.2	0.9978
Week 6	−6.0	−6.5	0.3024
Week 7	−6.7	−7.4	0.1530
Week 8	−7.4	−7.7	0.5471
Endpoint	−7.0	−7.1	0.7626
LSM change from baseline in CGI-S score			
Week 1	−0.2	−0.2	0.4497
Week 2	−0.5	−0.5	0.9625
Week 4	−0.7	−0.8	0.1467
Week 6*	*−0.9*	*−1.2*	*0.0226*
Week 7	−1.1	−1.3	0.0757
Week 8*	*−1.2*	*−1.5*	*0.0159*
Endpoint*	*−1.1*	*−1.3*	*0.0320*
Proportion of CGI-S responders, *n* (%)^a^			
Week 1	4 (2)	4 (2)	0.9939
Week 2	15 (8)	16 (8)	0.8927
Week 4	28 (15)	36 (20)	0.2585
Week 6	44 (26)	56 (33)	0.1350
Week 7	55 (32)	68 (40)	0.1031
Week 8	66 (40)	84 (50)	0.0516
Endpoint	67 (34)	86 (44)	0.0503
Week 4*	*5.3*	*7.7*	*0.0113*
Week 8**	*11.4*	*15.2*	*0.0012*
Endpoint**	*10.4*	*13.5*	*0.0066*

*CGI-S* Clinical Global Impression of Severity of Illness, *GAF* Global Assessment of Functioning, *IDS-C*_*30*_ 30-Item Inventory of Depressive Symptomatology–Clinician-Rated, *LSM* least-square mean, *QIDS-C*_*16*_ 16-Item Quick Inventory of Depressive Symptomatology–Clinician-Rated *P<0.05. **P<0.01.

^a^The denominator for calculating the percentages at each visit is the number of patients with a non-missing value at that visit. A responder is a patient with a decrease of at least 2 points in severity from baseline in CGI-S rating for depression. The *P* value for the treatment comparison is from a Cochran-Mantel-Haenszel test, stratified by concurrent mood-stabilizing medication and region of the world

There were numeric, but not statistically significant, findings favoring adjunctive armodafinil versus adjunctive placebo on the LS mean ± SEM change from baseline to endpoint in IDS-C_30_ total score (−18.2 ± 1.23 vs −17.1 ± 1.23), QIDS-C_16_ total score (−7.1 ± 0.49 vs −7.0 ± 0.49), and IDS-C_30_ response at endpoint (49 vs 41 %). There were no statistically significant differences between groups in the proportion of CGI-S responders at any assessment point.

### Safety

Adjunctive armodafinil 150 mg/day in bipolar I depression was generally well tolerated. Overall, 89 (45 %) patients receiving adjunctive armodafinil and 71 (36 %) patients receiving adjunctive placebo experienced ≥1 AE. Of these, 53 (27 %) AEs with adjunctive armodafinil and 32 (16 %) with adjunctive placebo were considered treatment-related. Only 2 AEs were observed at a rate exceeding 5 % in either treatment group: headache in 29 (15 %) with adjunctive armodafinil vs 15 (8 %) with adjunctive placebo and nausea in 12 (6 %) with adjunctive armodafinil vs 7 (4 %) with adjunctive placebo. The majority of AEs were mild (in 53 (27 %) vs 47 (24 %)) or moderate (in 32 (16 %) vs 20 (10 %)) in severity for adjunctive armodafinil vs adjunctive placebo, respectively.

Rates of AEs causing withdrawals and incidences of SAEs were similar between groups. Withdrawals due to AEs occurred in 7 (4 %) patients taking adjunctive armodafinil and 10 (5 %) patients taking adjunctive placebo. Serious AEs occurred in 5 (3 %) patients taking adjunctive armodafinil and 6 (3 %) patients taking adjunctive placebo; no single SAE occurred in >1 patient. No deaths occurred in either treatment group. At study endpoint, there were no clinically significant differences in mean changes from baseline in serum chemistries, lipid profiles, and hematologic or urinalysis parameters noted between the two treatment groups. There were no clinically significant changes in vital signs or electrocardiograms in either treatment group.

There were few changes from baseline to endpoint in suicidal ideation and behavior as assessed by C-SSRS-SLV. In the adjunctive armodafinil group, 1 patient had an SAE coded using the Medical Dictionary for Regulatory Activities preferred term of “bipolar I disorder” (specifically, exacerbation of bipolar disorder (mixed episode with psychotic symptoms)), accompanied by a suicide attempt, with no substantive threat to life; both SAEs resolved with no residual effect. One patient taking adjunctive placebo had an SAE of suicidal ideation, which resolved without any sequelae.

Mean changes in YMRS, HAM-A, and ISI total scores from baseline to endpoint were statistically similar with adjunctive armodafinil vs adjunctive placebo, respectively, as follows: YMRS (−0.9 vs −1.0), HAM-A (−4.3 vs −4.2), and ISI (−7.1 vs −7.0).

The following AE rate differences were observed between adjunctive armodafinil and adjunctive placebo, respectively: emergence of hypomania (0 (0 %) vs 1 (<1 %)), anxiety (8 (4 %) vs 5 (3 %)), insomnia (6 (3 %) vs 4 (2 %)), sedation/somnolence (2 (1 %) vs 2 (1 %)), and ≥7 % weight gain (4 (2 %) vs 9 (5 %)).

### Discussion

There is a compelling need for effective, well-tolerated treatments for bipolar I depression, for use adjunctively or as monotherapy. Despite the clinical practice of adding adjunctive treatments to ongoing maintenance medications when an episode of bipolar I depression emerges, there is little adequately controlled evidence to inform such clinical decisions. In fact, only one agent, lurasidone, has been approved by the FDA for adjunctive use (combined with lithium or valproate) for bipolar I depression (Latuda [package insert], 2013).

A phase 2 study (Calabrese et al., 2010) and two subsequent phase 3 studies (Calabrese et al., 2014; Ketter et al., 2015) previously evaluated the efficacy and safety of adjunctive armodafinil treatment for depressive symptoms in bipolar I disorder, both using the same primary efficacy measure (IDS-C_30_). Conflicting findings from the first two phase 3 studies warranted additional research. In the current study, the adjunctive use of armodafinil 150 mg/day for bipolar I depression provided a numerical but nonsignificant difference vs placebo on the primary outcome (reduction of depressive symptomatology as measured by the IDS-C_30_ total score). The negative primary outcome result of this study was similar to that of the similarly designed second phase 3 study (Ketter et al., 2015) and did not support or confirm the statistically significant primary outcome finding from the initial phase 3 study (Calabrese et al., 2014). However, in the current study (like the first study and unlike the second study), adjunctive armodafinil 150 mg/day compared with placebo yielded statistically significant benefits for several secondary outcomes, including the proportion of IDS-C_30_ responders at weeks 6, 7, and 8, proportion of IDS-C_30_ remitters at week 8 and endpoint, and mean change from baseline in IDS-C_30_ total score at week 7. In addition, mean change from baseline in the CGI-S rating for depression (indicating improvement in depressive symptomatology) was statistically significant at weeks 6 and 8, and at endpoint, and mean change from baseline in the GAF score (indicating improvement in patient functioning) was statistically significant at weeks 4 and 8, and at endpoint. In all 3 studies, adjunctive armodafinil was consistently well tolerated, with no clinically relevant differences vs adjunctive placebo for the emergence of hypomania, insomnia, anxiety, sedation/somnolence, or ≥7 % weight gain.

Some patients with bipolar disorder may be candidates for combination therapy to manage manic, hypomanic, mixed, depressed, and/or cycling related mood symptoms. In contrast to most regulatory non-monotherapy bipolar disorder clinical trials, which limit adjunctive treatments to lithium, valproate, and only very recently lamotrigine or one atypical antipsychotic, this study allowed 29 different combinations of mood-stabilizing treatments prior to study randomization (see Table 2). The assay sensitivity (i.e., drug–placebo separation) of this study was potentially limited by what was identified a priori as a study design strength (i.e., potential generalizability and community translation). A second challenge overall is the design of a study for a novel compound that is not considered a “mood stabilizer” where the trial design template has conventionally been designed for atypical antipsychotic mood stabilizers (i.e., lurasidone, olanzapine/fluoxetine, and quetiapine).

## Conclusions

In this 8-week study marked by significant therapeutic heterogeneity, adjunctive armodafinil 150 mg/day yielded a numerically greater, but not statistically significant, improvement on the primary efficacy outcome measure compared with placebo in treatment of bipolar I depression. Several secondary outcomes favoring armodafinil at endpoint, including a significantly higher remission rate, and paralleled by significant global and functional improvements rated by clinical investigators, indicate that adjunctive armodafinil provided a reduction of depressive symptoms in a subset of patients with bipolar I depression vs placebo. Adjunctive armodafinil was generally well tolerated in this study, with rates of anxiety, insomnia, sedation/somnolence, and weight gain similar to those observed with adjunctive placebo.

This investigation adds to the evidence from two prior phase 3 studies of similar design, in which armodafinil 150 mg/day, adjunctive to protocol-allowed mood stabilizers, provided statistically significant improvement in at least some measures of depressive symptoms associated with bipolar I disorder (Study 3071; NCT01072929 and Study 3072; NCT01072630) (Calabrese et al. 2014; Ketter et al. 2015). Although armodafinil was generally well tolerated in this and prior phase 3 studies, the lack of statistically significant efficacy on the primary outcome in two of three studies has led to the discontinuation of the development program for adjunctive armodafinil in bipolar I depression. Additional research is warranted and necessary to better identify clinical predictors (e.g., atypical depressive symptoms, specific combinations of therapeutic agents) that would provide optimized and individualized therapeutics for bipolar depression.

## Abbreviations

AE: adverse event
CGI-S: Clinical Global Impression of Severity of Illness
C-SSRS-SLV: Columbia-Suicide Severity Rating Scale-Since Last Visit
GAF: Global Assessment of Functioning
HAM-A: Hamilton Anxiety Scale
IDS-C_30_: 30-Item Inventory of Depressive Symptomatology–Clinician-Rated
ISI: Insomnia Severity Index
LSM: least-square mean
MMRM: mixed-model repeated measures
QIDS-C_16_: 16-Item Quick Inventory of Depressive Symptomatology–Clinician-Rated
SAE: serious adverse event
SD: standard deviation
SCID-CT: Structured Clinical Interview for DSM-IV, Clinical Trials
YMRS: Young Mania Rating Scale
